# Editorial: How current and future approaches and research results contribute to evolve the field of vascularized composite allotransplantation

**DOI:** 10.3389/frtra.2026.1993324

**Published:** 2026-10-02

**Authors:** Martin Kumnig

**Affiliations:** Department of Psychiatry, Psychotherapy, Psychosomatics and Medical Psychology, Center for Advanced Psychology in Plastic and Transplant Surgery (CAPPTS), Medical University of Innsbruck, Innsbruck, Austria

**Keywords:** evaluation, follow-up, outcome research, patient centered medicine, psychology, psychosocial, team science, vascularized composite allotransplantation

## Introduction

Vascularized composite allotransplantation (VCA) has evolved remarkably since the first successful hand transplantation procedures in the late 1990s. Over the past twenty five years, the field has progressed from experimental microsurgical innovation toward a highly interdisciplinary discipline integrating reconstructive surgery, transplantation immunology, rehabilitation medicine, psychiatry, psychology, ethics, digital technologies, and regenerative medicine. Contemporary VCA procedures, including face, upper extremity, abdominal wall, uterine, and genitourinary transplantation, aim not only to restore anatomical structures and physical function, but also to improve embodiment, identity, dignity, autonomy, and social participation in patients affected by devastating tissue loss. Despite extraordinary technical progress, major challenges remain regarding lifelong immunosuppression, psychosocial adaptation, ethical proportionality, equitable access, and long term sustainability.

### The aim of this special issue

This Frontiers in Transplantation Research Topic, entitled “How Current and Future Approaches and Research Results Contribute to Evolve the Field of Vascularized Composite Allotransplantation,” was developed to critically examine how technological innovation, psychosocial care, ethical governance, and interdisciplinary collaboration collectively shape the future of VCA. Looking back on more than two decades of international experience, it has become increasingly evident that long term success cannot be measured solely through graft survival or technical outcomes. Instead, meaningful outcome assessment must also incorporate quality of life, psychosocial resilience, rehabilitation adherence, informed decision making, embodiment, and social reintegration. Collectively, the contributions included in this issue illustrate how VCA is transitioning from a technically driven experimental endeavor toward a mature, patient centered, and ethically reflective field within modern transplantation medicine.

### Statistics on this research topic

At the time of writing, this Frontiers Research Topic has generated considerable international visibility and scholarly interest. According to the Frontiers in Transplantation website, the Research Topic achieved more than 8.589 total views and downloads, including over 7.291 article views and 1.848 article downloads. The topic further brought together an international group of more than 30 contributing authors from major transplantation and reconstructive surgery centers worldwide. These metrics illustrate the growing scientific and clinical interest in the psychosocial, ethical, and technological dimensions of VCA and highlight the importance of interdisciplinary collaboration within this rapidly evolving field.

### Technological innovation and emerging translational approaches

Several contributions emphasize the accelerating pace of technological innovation in VCA. The bibliometric analysis by Njessi et al. provides an important historical perspective on the evolution of the field by examining the one hundred most cited publications in VCA. Their findings demonstrate that hand and face transplantation, immunological innovation, rehabilitation science, and ethical discourse have fundamentally shaped the scientific literature, while also highlighting the highly interdisciplinary character of contemporary VCA research.

Similarly, the systematic review by Chakradhar et al. synthesizes emerging technologies in immunomodulation, targeted drug delivery, tolerance induction, machine perfusion, biomarkers, and digital innovation. The authors demonstrate how localized tacrolimus delivery systems, hydrogel based immunosuppressive platforms, mesenchymal stem cell therapies, and mixed chimerism protocols may reduce the burden of lifelong systemic immunosuppression while improving graft survival and individualized care.

The translational study by Duru et al. further advances this technological trajectory by demonstrating successful twenty four hour *ex vivo* perfusion of vascularized composite allografts in a large animal limb model. Their findings suggest that prolonged extracorporeal preservation may significantly improve donor logistics, ischemia reperfusion injury mitigation, donor recipient matching, and broader geographic allocation possibilities. Such innovations may eventually reshape organ preservation strategies and improve global accessibility within VCA.

Additional contributions extend these discussions into specialized areas of transplantation medicine. D'Elia et al. review immunosuppressive management in uterine VCA and pregnancy, emphasizing the unique challenges associated with temporary grafts, maternal fetal immunology, and pregnancy related pharmacokinetic changes. Their review highlights the importance of individualized therapeutic monitoring, pregnancy compatible immunosuppressive agents, and future development of noninvasive rejection monitoring and tolerance inducing approaches.

### Psychosocial, developmental, and ethical dimensions

Importantly, this Research Topic consistently demonstrates that technological innovation alone cannot define successful VCA outcomes. The mini review by Kumnig et al. argues for a biopsychosocial technological framework integrating biomedical advances with psychosocial care, ethical reflection, and patient centered research. Because most VCA procedures are life enhancing rather than life saving, the ethical balance between functional benefit and long term medical risk remains central to clinical decision making.

Several contributions further emphasize the importance of psychosocial assessment and longitudinal support. Antas and Nyberg, drawing on the Finnish VCA experience, describe psychosocial suitability as a dynamic and multidimensional construct that extends beyond the absence of psychiatric illness. Their observations highlight psychosocial functioning, cognitive capacity, social context, and mentalization capacity as particularly important dimensions for preoperative evaluation and long term follow up.

McKenna et al. extend these ethical and psychosocial considerations to facial transplantation in emerging adulthood. They argue that individuals between eighteen and twenty five years of age represent a uniquely vulnerable yet potentially highly benefiting patient population. Although emerging adults may experience substantial improvements in identity formation and social integration following facial transplantation, ongoing cognitive development may complicate informed consent, adherence, and long term decision making. The authors therefore advocate for developmentally tailored psychosocial support strategies to optimize outcomes in this population.

## Conclusions

Collectively, the contributions within this research topic illustrate that the future of VCA depends upon integrating technological innovation with psychosocial resilience, ethical governance, individualized medicine, and interdisciplinary collaboration. Advances in immunomodulation, machine perfusion, digital technologies, and regenerative approaches continue to expand clinical possibilities. At the same time, patient centered care, psychosocial follow up, equitable access, and ethical reflection remain indispensable foundations for sustainable progress. The future evolution of VCA will therefore depend not only on technical achievement, but also on the field's capacity to support the complex psychological, social, and ethical realities experienced by recipients throughout their lifelong transplantation journey.

At the time of writing, this Research Topic has generated substantial international scholarly interest and has brought together contributors from transplantation surgery, reconstructive microsurgery, psychiatry, psychology, immunology, rehabilitation medicine, and bioethics. The diversity of disciplines represented throughout this issue reflects the increasingly collaborative nature of modern VCA research and clinical practice. Importantly, the articles collectively underscore that future progress in VCA will require harmonized international registries, standardized psychosocial outcome measures, long term follow up protocols, and ethically grounded frameworks for patient selection and informed consent. Continued collaboration between clinicians, researchers, ethicists, rehabilitation specialists, and patient advocates will be essential to translate scientific innovation into sustainable clinical care pathways that improve both graft related outcomes and long term quality of life for transplant recipients worldwide.

Together, these contributions provide an important roadmap for future interdisciplinary research, innovation, and responsible clinical implementation within VCA worldwide in coming years.

